# Solid Features Modification by the Reactor Selection and US Support during Reactive Crystallization

**DOI:** 10.3390/ma15217419

**Published:** 2022-10-22

**Authors:** Magdalena Stec, Piotr Maria Synowiec

**Affiliations:** 1 Department of Chemical Engineering and Process Design, Faculty of Chemistry, Silesian University of Technology, Strzody 7, 44-100 Gliwice, Poland; 2 Łukasiewicz Research Network, New Chemical Syntheses Institute, InorChem Research and Development Center, Sowińskiego 11, 44-100 Gliwice, Poland

**Keywords:** sonoprecipitation, calcium fluoride, ultrasounds, stirred tank reactor, static mixer

## Abstract

The use of materials requires adjusting their features to current applications/needs. In crystallization, the production methods leading directly to the product with pre-determined characteristics are being sought. The research focuses on the abilities of “shaping” the solid product (CSD, shape, form, etc.) and is based on experimental work carried out in the ultrasound (US)-assisted Koflo static mixer (STM). As the model reaction calcium fluoride precipitation has been used as a “common denominator” that complements the previous authors’ studies, providing comprehensive knowledge and a more general look at the mentioned problem. It has been shown that it is possible to obtain crystals with the desired characteristics; however, one should be aware of the used reactors’ limitations. The conscious selection of operating conditions, as well as US parameters (if they are used), is also essential. It has been revealed that the introduction of US to the STM only affects the turbulence intensity, but it doesn’t change the mixing profile. The kinetics of crystallization remain unchanged, but crystals are subjected to greater attrition. In the stirred tank reactors, one might significantly improve the homogeneity of the unit mixing distribution by the selection of the relative input power *ε_rel_* and, thus, affect the kinetics of crystallization.

## 1. Introduction

Demands regarding solid substances introduced to the markets are still rising. On the one hand, such a trend is dictated by the need to ensure a high quality of manufactured products, among others, due to environmental protection regulations, health care, defense of living organisms, etc. On the other hand, it is caused by the companies’ economic balance, on the basis of which they seek the reduction of processing step numbers. Therefore, any method, even approximate, allowing for the prediction of the required crystals’ characteristics before their production, based on the selection of operating conditions in a reactor, is in high demand.

Preliminary attempts to develop such a method had already been made by the authors in their earlier works [1,2,3]; however, in the light of the latest research, being a résumé to the previous studies, some important novelties and modifications were undeniably required to reach more universal descriptions and conclusions, respectively.

The long-term research dedicated to the reactive crystallization (precipitation), carried out by the authors previously [1,2,3], took the experiments in a stirred tank reactor (STR) with a turbine agitator in both silent and US-assisted conditions into account, as well as in a silent in-line reactor Koflo (Cary, IL, USA) with semi-circular static inserts (STM). However, to have a broader background, the experiments had to be continued with the use of the US-assisted Koflo STM. Such an attempt enables a more general look at the examined process, as, finally, all of the experimental works stand as one coherent whole. It will also allow us to understand and explain the equivocal role of ultrasounds introduced to reactors of different types.

It is also worth mentioning that the last series of experiments has never been published by the authors before.

As a model reaction, the precipitation of calcium fluoride (precipitated from ammonium fluoride and calcium nitrate solutions) has been used as a common denominator, combining all the experiments. The decision regarding the use of two completely different reactors types (STR and in-line STM) has been made, most of all, according to the important differences between the operation basis and the distribution of the unit mixing powers (the energy dissipation rates), which might have a substantial influence on the process kinetics, thus affecting the final product characteristics. As proven during CFD analyses [4], static mixers are characterized by an almost homogeneous distribution of the unit power input *ε*. Thanks to that, the micromixing effect stays at the same level in the entire reactor volume; hence, the appearance of areas with uncontrolled supersaturation is less likely. When the reactors with a mechanical agitator are taken into account, the situation is no longer so simple. The volume within the STR is divided into zones, differing from each other in the unit mixing power. The areas where the mixing is very weak, as well as so-called “dead areas”, are also present. The most intensive mixing zone is localized in the nearest area of the agitator’s blades; however, the absolute value of the local unit power input *ε_loc_* depends strongly on the type of the used mixer [5,6,7]. Therefore, one should be aware that not only does the selection of a reactor type matter, but the selection of an agitator should also be made consciously. Besides the above-mentioned, there are other pros and cons that should be taken into account during the selection of a reactor type. For example, in static mixers, the residence times are rather short, so their use is limited to processes in which the time of media contact is not very long. In practice, it means that, during the crystallization, static mixers would be useful for processes directed to the nucleation stage (not to crystal growth). What is more, the mixing time is inseparably associated with the fluid velocity and the device length, and it cannot be set apart from them. The transport of fluids/suspensions is occupied with high pressure drops, which makes STMs power-consuming devices. In turn, STR offers any residence time, regardless of the mixing conditions, so even long-hour mixing is possible to carry out.

Due to the fact that many of the manufactured products are obtained in the agglomerated form, effective techniques for reducing this phenomenon are also being sought. As one of the methods used, the literature shows the US assistance. It is also known that the introduction of ultrasounds to the reaction system may act on particles’ characteristics by changing their shape, reducing their size, narrowing their distribution, etc. [8,9,10,11]. During the authors’ research, carried out in the STR [3], it was observed that only properly selected US parameters may help to obtain the product with the defined features. Otherwise, they may contribute to the excessive destruction of primary crystals (attrition, breakage) and, therefore, the appearance of secondary agglomeration. Therefore, it is also of great importance to check whether the same behavior is observed in STMs, in which mixing characteristics are significantly different. What is also essential and should be recognized is the influence of US use in the reaction systems. Is their role limited only to the increase of turbulence intensity? Or maybe the effects of US are more complex? Finding the answers to the above-mentioned question would also broaden the existing knowledge.

In light of the presented information, the main aim of the discussed paper, based on the combination of the newest research with the previous results (treated as an inseparable whole), is to present a practical guideline facilitating the conscious decision about the selection of crystallizer type and operating conditions, as well as the necessity of US use, together with the choice of parameters, such as (*P_US_*, *f_US_*, *τ*) to adjust them to the solid product requirements.

## 2. Calculations’ Basis

As explained in detail in previous papers [1,2,3], for the common description of turbulence intensity in different types of reactors, the proposed equivalent Reynolds number (Equation (1)) has been used. As mentioned in the introduction, because of a non-homogenous mixing profile in STR, some modifications in the definitions of the unit power input (Equation (4)), as well as the equivalent diameter (Equation (6)), were absolutely necessary, as the previous calculation methods provided falsely overestimated values of *Re_eqv_*. That, in turn, translated into inconsistencies in the proposed crystal size prediction method, which had to be refined.

(1) $$Re_{eqv}=\varepsilon^{\frac{1}{3}}\cdot d_{eqv}{}^{\frac{4}{3}}\cdot \frac{\rho }{\eta \prime }$$

For the systems working in silent conditions, the unit power input was calculated as equal to the local unit mixing power (Equation (2)), as the turbulence was induced only by the mechanical agitator.

(2) $$\varepsilon =\varepsilon_{mix}\;\left[\frac{W}{kg}\right]$$

Because the distribution of *ε* in static mixers is very uniform [4], the average value was assumed as the local value and calculated as (Equation (3)):

(3) $$\varepsilon_{{}_{STM_{\;mix}}}=\varepsilon_{av}=\frac{\dot{V}\cdot \Delta p}{V_{STM}\cdot \rho }\;\left[\frac{W}{kg}\right]$$

When the research has been conducted in STR, the reagents have been dosed near the agitator’s blades to introduce them into the area with intensive mixing and assure high reaction efficiency. As known from the literature [5,6], the energy dissipation rate in the closest area of the stirrer may be assumed as several times greater than the average value, depending on the type of agitator used and the distance from the stirrer’s blades. In accordance with that, the local (active) value of *ε* in STR has been determined as (Equation (4)):

(4) $$\varepsilon_{{}_{STR_{\;mix}}}=X\cdot \varepsilon_{av}=X\cdot \frac{Ne\cdot d_{m}^{5}\cdot n^{3}}{V_{STR}}\;\left[\frac{W}{kg}\right]$$

where *X* [-]—conversion factor (generally *X* ≈ 4 ÷ 10), enabling the estimation of the *ε_loc_* value (close to the maximum one), depending on the type of the used agitator and the distance from the stirrer’s blades.

For a turbine stirrer, in the closest area of the blades, the value of *X* may be assumed equal to 10 [5,6].

The calculations of reactors’ equivalent diameters were based on Equations (5) and (6) for static mixers and the tank reactor, respectively.

(5) $$d_{eqv_{STM}}=\sqrt{\frac{4\cdot V_{STM}}{\pi \cdot L_{m}}}\;\left[m\right]$$

(6) $$d_{eqv_{STR}}=\sqrt[{3}]{V_{act}}\;\left[m\right]$$

The procedure used for the determination of the active volume of STR, considered as the partial volume in which the maximum value of the unit mixing power input *ε_mix_* is observed, is as follows (Equations (7)–(9)).

(7) $$V_{act}=\frac{\pi }{4}\cdot \left(d_{act}{}^{2}-d_{m}{}^{2}\right)\cdot h_{m}\;\left[m^{3}\right]$$

The active diameter, as one of the parameters representing the active volume, may be calculated as:

(8) $$d_{act}=Y\cdot d_{m}\;\left[m\right]$$

where *Y* is the coefficient used for the evaluation of the active diameter on the basis of a mixer’s diameter *d_m_*, depending on the used agitator’s type.

Taking into account the relevant literature [5,6,7] and the used mixer type–turbine stirrer, it was determined that the area where $\varepsilon_{STR_{mix}}=10\cdot \varepsilon_{av}\;\left[\frac{W}{kg}\right]$ includes:

(9) $$d_{act}=1.13\cdot d_{m}\;\left[m\right]$$

One should mention that the method previously described by the authors, used for the calculation of the STR equivalent diameter $\left(d_{eqv_{STR}}=\sqrt{D^{2}-d_{m}{}^{2}}\right)$ [1,2,3], turned out to be inappropriate, as it referred to the entire volume of a reactor, instead of an active volume, which provided a greatly overestimated value of turbulence intensity represented by the equivalent Reynolds number. Due to the existence of areas with insufficient mixing, as well as dead zones, such an approach was imprecise, as high-intensity mixing is present only in the nearest area of the mixer’s blades.

In turn, when US-assisted precipitation methods were used, for the turbulence intensity induction, both the mechanical mixing and ultrasounds present in the system were responsible. Due to that, the total energy dissipation rate might be calculated as Equation (10):

(10) $$\varepsilon =\varepsilon_{mix}+\varepsilon_{US}\;\left[\frac{W}{kg}\right]$$

The unit power input caused by ultrasounds has been determined by the use of Equation (11).

(11) $$\varepsilon_{US}=\frac{P_{US}}{V_{UB}\cdot \rho }\;\left[\frac{W}{kg}\right]$$

## 3. Materials and Methods

The precipitation of calcium fluoride, as a model reaction used in all of the experiments, has been carried out by the use of ammonium fluoride solution (0.244 M) and calcium nitrate solution (0.122 M), Equation (12), from a supersaturated solution, in which the relative supersaturation *σ* was equal to 157 [4].

(12) $$2NH_{4}F\;+\;Ca{\left(NO_{3}\right)}_{2}\;\to \;CaF_{2}+2NH_{4}NO_{3}$$

In the laboratory setup used for the experimental research (Figure S1 in the supplement), as the ultrasounds generator, the US-bath was used (FALC, Treviglio, Italy, volume 10 L, frequencies 40/59 kHz, maximum power 300 W with the automatic adjustment in the range of 0–100% *P_US_*). When the process in silent conditions was tested, the ultrasonic bath stayed turned off.

From the feed tanks, substrate solutions were dosed by the use of pumps (Masterflex (Vernon Hills, IL, USA) driver with two Easy–Load heads, measuring range (from 0.001 mL to 2900 mL) to the Koflo STM (or STR in previous studies)) with the same volumetric flow rate. The substrates were introduced to the reactor into the inlet cross-sectional area in a perpendicular way. In turn, when experiments with STR were carried out, the substrates were dosed in the reactor in the nearest area to the mixer’s blades, to reach a high turbulence intensity zone and assure high reaction efficiency. Each reactor was placed inside the ultrasonic bath, which was turned on during the experiments using the US-assisted methods and turned off during the tests under silent conditions. The measurements have been started after a steady state was reached. The liquid–solid suspension obtained during the research was directed to the storage tank. To ensure operational safety and to enable continuous control of the process, the laboratory setup was equipped with control valves and thermostats (Julabo MA-12 (JULABO GmbH, Seelbach, Germany), temperature range (−20–200) °C, accuracy ±0.01 °C, maximum flow rate up to 16 L/h).

Solid particles were next separated from the mother liquor using vacuum filtration (sometimes in combination with the use of membrane filters preventing the finest crystals from getting into the filtrate), washed by the use of analytical grade ethanol, dried at the temperature of 60 °C, and next directed to analyses.

The laser particle size analyzer, Malvern (Malvern Panalytical Ltd., Malvern, UK) Mastersizer 3000 (measuring range 0.01–2100 μm, precision >0.5%), has been used to determine the CSD of the collected product. The measurements were carried out in a wet Hydro EV module, with the use of water as a dispersant. During the CSD measurement cycle (3 measurements), the first measurement was carried out without any additional support of a built-in US probe, and the next two were carried out with the additional support of US (sample sonication time 60 s, stabilization after sonication: 60 s, the power of US: 80% *P_nom_* (*P_nom_* = 40 W) and 100% *P_nom_*, respectively) to observe the suspension behavior with time.

SEM observations, showing the shape of crystals and their form (single crystals or agglomerated clusters) have been performed by the use of the scanning electron microscope Phenom ProX (ThermoFisher Scientific, Waltham, MA, USA), working with a back-scattered electron detector (BSD) (acceleration voltage 15 kV) or Hitachi (Hitachi, Tokyo, Japan) TM 3000 (in the case of previous studies, relating to the use of STR in silent conditions).

The information about the physical properties of the liquid reactants has been obtained by the use of standard equipment, such as a densimeter (Anton Paar (Anton Paar GmbH, Graz, Austria) with the accuracy ±5 × 10^−5^ g/cm^3^) and a viscometer (Visco Lab (PAC L.P., Houston, TX, USA) 4000, precision ±1.5%).

The properties of reactants used (at the process temperature *t* = 20 °C), as well as the dimensions of the used reactors, are presented in Tables S1 and S2, attached as supplementary materials.

The collection of working conditions, at which all of the experiments have been carried out, is presented in the Supplementary Materials, Tables S3–S6, for both types of reactors used (working in silent and US-assisted conditions).

## 4. Results and Discussion

To facilitate the selection of precipitation operating conditions, as well as the selection of the crystallizer type, in such a way as to obtain the solid particles with the predefined characteristics, a comprehensive guideline, based on the results obtained during long-time research, combined with the latest ones, carried out in the US-assisted STM, has been presented in this section.

### 4.1. Agglomeration Phenomenon Limitation/Induction

The occurrence of the agglomeration phenomenon may have both positive and negative aspects. On the one hand, the formation of large particles’ clusters facilitates and speeds up the filtration operation (that might be the slowest, process-limiting stage, due to the time required for solid–liquid separation). On the other hand, it might have a negative impact on the product quality, as the traces of mother liquor that binds the primary particles together reduces the product purity, hence degrading the final quality. That is why the decision regarding whether the agglomeration phenomenon would be useful depends on the final product designation.

**Figure 1:**
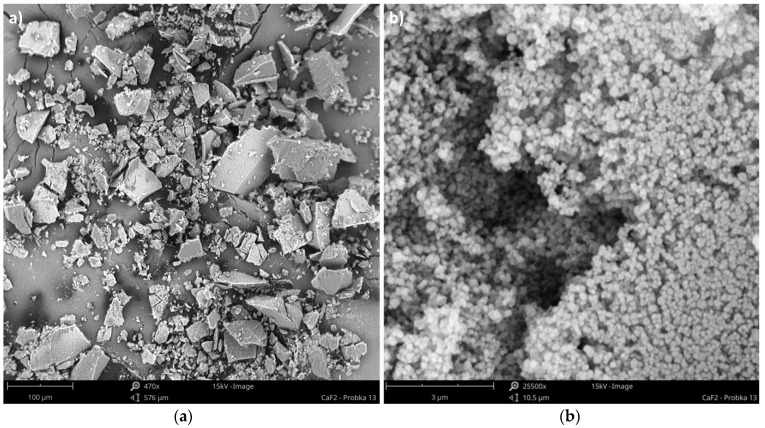
SEM images of CaF_2_ particles precipitated in the Koflo static mixer—silent conditions. Operating conditions: *ε_mix_* = 4.9 W/kg, *Re_eqv_* = 3994; (**a**) magnification 470×, size bare: 100 μm; (**b**) magnification 25,500×, size bare: 3 μm.

Agglomerated particles are quite easy to obtain, especially when small-sized particles (less than a few microns) are precipitated. One might use both reactor types: in-line with static inserts, as well as STRs. In STMs, due to the increased number of very intense crystal-motionless inserts interactions, compressive forces so great that the primary particles do not constitute single ones anymore, but are pressed together, forming a product with a size of up to 400 μm (depending on the fluid dynamic conditions), which looks like the crushed filter cake—Figure 1a. In the authors’ reports [1,2,4], they were taken, at first as, primary particles. Re-analysis of the obtained solid by the use of new methods and more accurate devices (new laser particle analyzer, Malvern Mastersizer 3000, instead of worn-out Fritsch Analysette, scanning electron microscope, Phenom ProX, with a magnification up to 150,000, instead of Hitachi TM 3000, with a magnification up to 30,000) has revealed the agglomerated and compressed character of the solid—Figure 1b.

The use of STR equipped with a turbine stirrer also resulted in obtaining agglomerated structures (made of primary particles with a mean size up to 2–3 μm); however, their sizes stayed within the range of 50–60 μm (published as Figures 8–10 in [1]). The size of the particles’ cluster increased slightly with an increasing value of the stirrer’s speed, as the limit value, in the analyzed range of *Re_eqv_*, causing agglomerates breakage that has not been reached (the tensile strength of agglomerates was higher than the destruction forces). As the compressive forces caused by the use of a mechanical stirrer were much smaller than the ones in static mixers, agglomerates obtained in STR have shown more incompact character.

**Figure 2:**
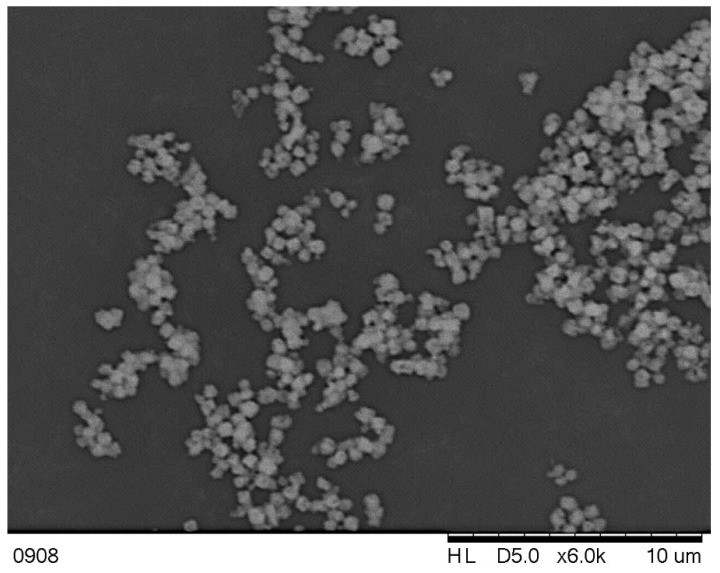
SEM images of CaF_2_ particles precipitated in STR—silent conditions. Operating conditions: *ε_mix_* = 0.4 W/kg, *Re_eqv_* = 3022, *τ* = 6 s, $\dot{V}$= 150 L/h (Hitachi TM 3000, magnification 6000×, size bare: 10 μm).

In turn, when a high-quality product is in demand, then the contribution of the agglomeration phenomenon should be limited. In such a case, the STR, as a reactor allowing us to obtain single particles directly in the process, is applicable. To achieve the intended goal, one may choose the solution from the presented methods:

- Increase the unit power of mechanical mixing (*ε_mix_*) to a value, at which the agglomerated structures will fall apart into the primary particles (so that the tensile strength of clusters is lower than the destruction forces);
- Increase the volumetric flow rate of reactants to make the turbulence in the reaction system more intense, reduce the thickness of a viscous layer on the crystals surface acting as a binder, and limit the time (as so as the number) of crystal-stirrer and crystal–crystal collisions (Figure 2);
- Use the ultrasounds-assisted methods of precipitation.

The first option is a bit inconvenient, as it requires a number of experimental tests that allow for finding the proper stirrer’s unit power input that, on the one hand, would break the agglomerates into primary particles, but on the other hand, would not cause the fragmentation of primary particles, leading to the secondary agglomeration. What is also very important is, even if such a value would be designated, sometimes it may not be applicable in practice (i.e., too high values may result in an unstable vortex flow, together with an unjustified increase in energy consumption, etc.).

The use of the second option is also limited, as one should check experimentally if the increased turbulence in the reaction system would not result in increased destruction forces, causing the breakage of primary particles. What is more, the increased flow rate of substrates must also be justified by the production capacity.

The third method, namely the use of US assistance during the precipitation in STR, might be a useful tool for limiting the contribution of agglomerated clusters, however, only if the US parameters (i.e., US power *P_US_*, US frequency *f_US_*, sonication time) are selected consciously (taking into account the impact of each parameter on particles characteristics). The key parameter that has the greatest influence on the agglomeration phenomenon is ultrasonic power. The ultrasonic power affects the number of cavities, the greater the power, the greater the number of vapor bubbles, hence much more intense turbulence, due to their violent implosions. The optimum value (in the considered research between 225 and 245 W, which corresponds to *ε_US_* = 22.3 ÷ 24.3 W/kg, respectively) must be found, as too low values would be insufficient to break all of the agglomerated structures and too high values would break not only the agglomerates, but also the primary particles, leading, at first, to sono-fragmentation, and next to the secondary agglomeration of the particles’ destroyed parts.

In the case of US-assisted methods used in combination with STMs, the reduction of the agglomeration phenomenon, among others, due to a compressed character of the obtained solid, was possible only after the process when a liquid–solid suspension was moved to the additional US-generator (beaker with the ultrasonic probe). To avoid agglomerates in the dry product, as well as the proper selection of suspension separation method (as well as accompanying steps, i.e., washing) is of high importance.

### 4.2. Nucleation Intensity Stimulation

Generally, in the case of fast heterogeneous ionic reactions, the control or stimulation of the nucleation intensity is not an easy task. Such a reaction type generates a high level of supersaturation, which may mainly initialize the primary nucleation. When the process is supported by US, the genesis of the phenomenon becomes even more complex.

The ultrasound assistance, represented mainly by the parameters such as US power, frequency, and time of exposure may affect the dominant stage in the crystallization processes. The selection of *f_US_* from the “low-frequency region”, which is between 20 and 100 kHz, results in a nucleation intensity increase [9]. In turn, higher frequencies (200 kHz to 2 MHz) may enhance crystal growth [12]. What is also very important, and has already been described by the authors in the previous paper, it that it is not only the low frequency that affects the nucleation intensity. The key parameter is the relation between the energy induced in the system by ultrasounds *ε_US_* and the energy introduced by a mechanical stirrer *ε_mix_*. The mentioned relation is represented by a dimensionless relative input power *ε_rel_*—Equation (13).

(13) $$\varepsilon_{rel}=\frac{\varepsilon_{mix}+\varepsilon_{US}}{\varepsilon_{mix}}$$

**Figure 3:**
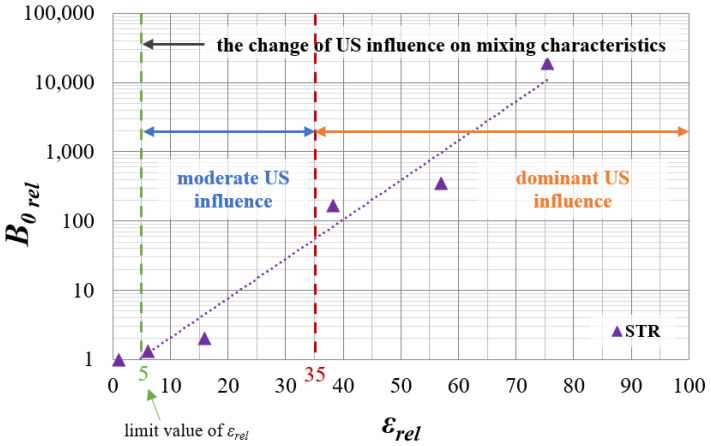
The relative nucleation intensity *B*_0*_rel_*_ as a function of the relative input power *ε_rel_* in the STR.

As it turns out, the distribution of the unit mixing power in the reactor used is also extremely important, which is presented in Figure 3. In the mentioned chart, the nucleation rate has been presented as a relative value, in an analogical way, to *ε_rel_*—Equation (14).

(14) $$B_{0{\;}_{rel}}=\frac{B_{0}{}_{{}_{mix}}+B_{0}{}_{{}_{US}}}{B_{0}{}_{{}_{mix}}}$$

The values of $B_{0_{mix}}$ and $B_{0_{US}}$ have been calculated in a simplified manner—Equation (15), taking the residence time, instead of nucleation time, into account, as in the considered cases, the experimental determination of *τ_ind_* (approx. 10^−9^) was very difficult. One should be aware of the fact that the calculations were not based on the MSMPR model, as it is not applicable in the considered cases (among others, due to the agglomeration, attrition, the use of different reactor types representing different mixing models, the additional support of US, etc.).

(15) $$B_{0}=n_{0}\left(\frac{L_{mean}}{\tau }\right)\;\left[\frac{1}{m^{3}\cdot s}\right]$$

The nuclei number density per unit volume *n*_0_ has been taken from the population density distribution *n(L)*, Equation (16), determined on the basis of data obtained directly from the laser particle analyzer.

(16) $$n\left(L_{i}\right)=\frac{w_{i}\cdot \phi }{k_{v}\cdot L_{i}^{3}\cdot \Delta L_{i}}\;\left[\frac{1}{m^{4}}\right]$$

In the case of the STM use, due to the existence of such phenomena as channeling or by-passing, the real residence time *t_m_* is not equal to the residence time *τ* (calculated as the ratio of the device volume to the volumetric flow rate). That is why, in Equation (15), *t_m_* calculated on the basis of RTD curves, Equation (17) [13], has been used. In the tank reactor *t_m_* = *τ*, which is why *τ* has been used.

(17) $$t_{m}=\int_{0}^{\infty }t\cdot E(t)dt\;[s]$$

More detailed information about the calculation of nucleation intensity in both reactor types, in silent and US-assisted conditions, may be found in the authors’ previous papers [2,3,4].

From the presented, Figure 3, which is based on the new, much deeper analysis of the previous data, it can be observed that the introduction of US to the STR may result in the change of the unit power input profile if the value of *ε_rel_* exceeds the limit of 5. Namely, when the unit power of US was about 4 times greater than the unit power of mechanical mixing, the assistance of the US increased, as well as the turbulence intensity; therefore, the area of high mixing broadens. One might suggest that, for 5 ≤ *ε_rel_* ≤ 35, the system stayed within the moderate influence of US, as the distribution of *ε* had been improved; however, it was not uniform yet. Due to that, only a small increase in the relative nucleation intensity *B*_0_ (up to 60–70 times greater) was observed, as in the reactor, the areas of insufficient mixing were still present. The situation was reversed when the system moved to the dominant influence of US (for *ε_rel_* > 35), which tended to align the energy dissipation profile that finally became more homogeneous. Due to that, the nucleation started taking place in the whole reactor volume, which caused a significant increase in *B*_0*_rel_*_ (in the considered research from about 100 to 10,000).

In the STM, such a trend was not visible, as the distribution of unit mixing powers is very close to homogeneous, even without the addition of US. It means that, in such a reactor type (in-line) ultrasound will act only on the turbulence intensity increase, without the change of mixing characteristics. Hence, no significant changes in the relative nucleation rate were observed (for *ε_rel_* in the range of 10–150 the increase of *B*_0*_rel_*_ was equal to 1.2–1.4 only, and for *ε_rel_* > 150, *B*_0*_rel_*_ stayed at a constant level of about 1.4 only).

### 4.3. Particle Size Distribution Directed to Mono- or Polydispersed Product

The number of processing stages required to obtain the product with the desired features, which, in turn, translates into product yield and operating costs, depends not only on the form of the product, namely agglomerated structures or single crystals, but also on the spread of CSD of the precipitated particles. On the basis of the presented criterion, one may divide the populations of crystals into the collection of polydispersed particles and close to monodispersed ones. Monodispersed products are in high demand, as their use provides many benefits, i.e., there is no need to sieve the product to separate the particles of different sizes, which then are milled or granulated to fit the desired final size. The dissolution time of similar-sized particles was comparable, which is particularly important during the production of pharmaceuticals or fertilizers, and the structure of pills produced from compressed monodispersed particles was much more uniform and, thus, better digestible, etc.

Monodispersed solid products may be obtained in a variety of ways, and the decision on which one should be used will be determined, most of all, by the required mean crystal size, as well as information regarding whether the presence of agglomerates is acceptable. 

**Figure 4:**
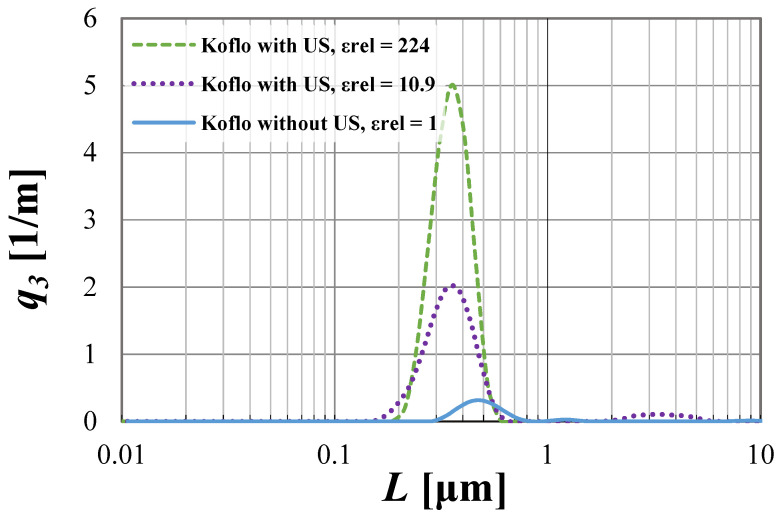
Particle density distributions of crystals obtained for different values of *ε_rel_* by the use of Koflo STM.

**Figure 5:**
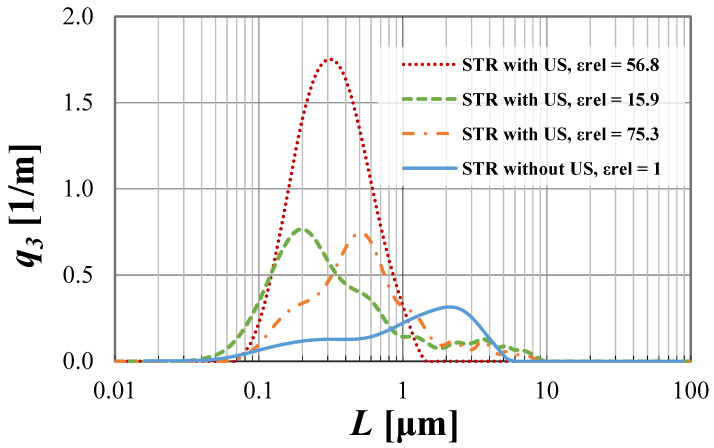
Particle density distributions of crystals obtained for different values of *ε_rel_* by the use of STR.

The possibilities of CSD limitations are presented in Figure 4 and Figure 5, with a detailed discussion.

From the presented Figure 4 and Figure 5, one may observe that the use of US-assisted precipitation might be a useful tool leading to the production of monodispersed solid products. However, one should be aware that the application of US to the systems differing from each other with the used reactor type (namely STR or STM) may reveal various beneficial aspects. In the authors’ opinion, the explanation of a very different influence of US on the spread of crystals’ populations obtained in static mixers and STR lies within the characteristic profile of the energy dissipation rate. As revealed during CFD analysis [4], the distribution of unit mixing powers in STMs is close to homogeneous; therefore, the introduction of US only impacts the turbulence intensity—it does not significantly change the *ε_mix_* profile. That is why the increase in the relative unit input power *ε_rel_* (whether by the increase of *ε_mix_* or by *ε_US_*), for the presented conditions, does not lead to significant changes, as observed in the STR. The dominant particle size stays at the same level, and the number of clustered particles being broken lessens, as most of the grains are single particles of primary size—Figure 4. However, one should be aware that a further increase of *ε_rel_* may, at first, be the cause of primary particles’ destruction, and next the secondary sono-agglomeration. In turn, in the silent STR, the distribution of *ε_mix_* is diversified, and one may distinguish the zones with high mixing intensities (near the blades of a mechanical stirrer), as well as the ones with moderate, weak, or even a lack of mixing [5,7]. Here, the addition of ultrasounds to the system results in both increased turbulence and, most of all, the alignment of mixing powers’ distribution. As presented in Figure 3, in the US-assisted STR, when the limit value is exceeded, one may see a change of mixing characteristics, depending on the applied relative input power *ε_rel_*. The contribution of ultrasounds may be moderate, with a partial alignment of the energy dissipation rate for 5 ≤ *ε_rel_* ≤ 40, or dominating, in which the distribution is close to homogeneous (for *ε_rel_* ≥ 40). Such an observation is of high importance, as it allows us to minimize, or even eliminate, the disadvantages of STR resulting precisely from the uneven profile of unit powers and to take full advantage of its benefits—most of all the possibility of selecting the mixing conditions and the residence time, independently of each other (what is impossible in in-line STMs). The obtained results (Figure 5) well-illustrate the above conclusions. For STR working in silent conditions, the collected product contained agglomerated structures of a mean size of ca. 1.1 μm. When US were added to the system, however, the relative input power (*ε_rel_* = 15.9 W/kg) was in the range of moderate US assistance, the chart was shifted towards smaller particle sizes, and the mean particle size was significantly decreased (*L_m_* = 0.2 μm). It means that the introduction of US caused the initiation of agglomerates breaking; however, the value of *ε_rel_* was insufficient to eliminate all of them (what is seen as a tail in the *q*_3_ curve). In turn, when the value of *ε_rel_* exceeded 40 (in the presented case *ε_rel_* ≈ 57 W/kg), we get into the area of the dominating influence of US, where all of the biggest agglomerates were destroyed, and the obtained product was closed to a monodispersed one. In the carried research, a further increase of *ε_rel_* (values greater than 65) caused particles’ fragmentation, and secondary sono-agglomeration occurred, as expected (visible as a pre-tail in *q*_3_ chart—Figure 5).

**Figure 6:**
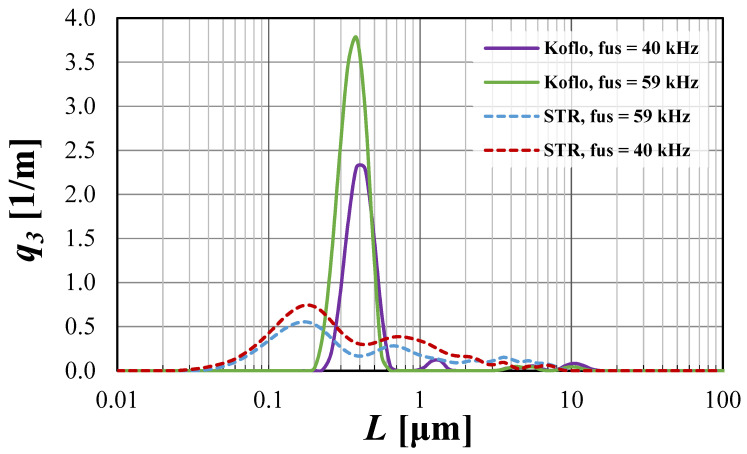
The influence of US frequency on particle density distribution *q*_3_ for both examined reactor types.

**Figure 7:**
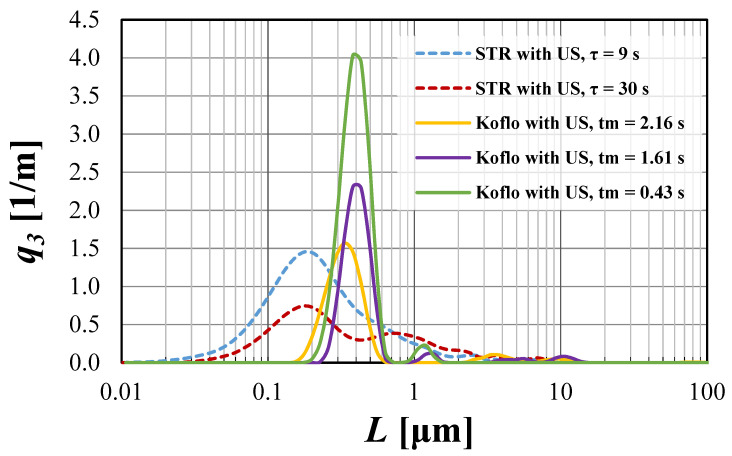
The influence of residence time on particle density distribution *q*_3_ for both examined reactor types.

Things to keep in mind, as well, are the parameters of US (such as power, frequency, and sonication time), which should be selected with caution. The proper selection of the ultrasonic power seems to have a crucial impact on the particles’ characteristics, but US frequency and sonication time (equal to the residence time in the carried research) are not without significance—Figure 4, Figure 5, Figure 6 and Figure 7.

Taking into account the influence of ultrasonic power on the particle size, one should point out that the character of the curves obtained for different values of *ε_US_* are quite similar to those presented in Figure 4 and Figure 5 (attached in the supplementary materials as Figure S2).

Ultrasonic power *P_US_* impacts the number of gas bubbles created in the reaction system, due to the acoustic cavitation phenomenon. The higher the power is, the greater number of voids created, and a higher turbulence intensity is observed, due to their violent implosions [10]. Generally, too low value of *P_US_* may not be enough to break all the agglomerated structures, and in turn, too high values may result in primary particles’ destruction (so-called sono-fragmentation), and then unwanted secondary agglomeration may appear. It was clearly visible, particularly when STR was used—Figure 5 (unbroken agglomerates are shown as a tail behind the green curve, and secondary sono-agglomerates made of tiny particles’ fragments are represented by a tail before the blue curve). Finding an optimum value is, therefore, very important. On the basis of the presented information, one may see that the ultrasonic power on the level of about 225 W (ca. *ε_US_* = 22.3 W/kg) allows for obtaining the product with the narrowest distribution (taking into account the analyzed values)—which is evidenced by the greatest maximum values of *q*_3_; however, the differences between the products collected from the STM and STR are visible. The use of the Koflo static mixer (*P_US_* = 225 W, *ε_US_* = 22.3 W/kg) led to the formation of a particles’ population, whose size was close to the dominant value (coefficient of variation *CV* was equal to 19.6%). It means that the obtained product may be assumed as an almost monodispersed one. When STR was used (*P_US_* = 225 W, *ε_US_* = 22.3 W/kg) in the population, there were particles with sizes in the range of 0.07 μm up to 1.05 μm, and a *CV* = 60.3%. The presented *CV* value was much higher than the one obtained in Koflo STM, so it can be suggested that the use of US-assisted static mixers would be a better solution if the product particles should be of the same (or very similar) size. However, if STR is the only option, one could consider a very slight increase of *P_US_* (bearing in mind that, in 300 W negative crystals’, destruction was observed) to find the direct optimum value that would narrow the distribution a bit more.

Coefficient of variation (CV) has been calculated on the basis of the presented equation [4]:

(18) $$CV=\frac{L_{84}-L_{16}}{2\cdot L_{50}}\cdot 100\;\left[\%\right]$$

If it is about the influence of ultrasonic frequency, then in the processes focused on the nucleation stage, in which precipitation is included, one should run the process in the so-called “low-frequency region”, which includes values between 20 and 100 kHz. As known from the literature [9,14,15], the ultrasonic frequency impacts the bubble size, namely the lower frequency results in the formation of bigger bubbles, which implode in a much more violent way, strongly increasing the turbulence; in turn, higher frequency leads to the formation of smaller cavitation voids, in which the implosion is not as strong. That is why, if an additional intensification of turbulence in the system is required (for instance, when further increasing the ultrasonic power is impossible or uneconomical), one may use lower values of frequency; however, it should be selected from the “low-frequency region”. In the described research, frequencies of 40 and 59 kHz have been examined, and in both reactor types, no significant changes between particles’ density distributions have been reported—Figure 6. It was caused by a minimal difference between the tested values and is consistent with the other researchers’ paper [16]. Comparing the curves obtained by the use of STM and STR, it might be seen that the combination of both ultrasonic frequency and power is also very important for obtaining a monodispersed product. As explained previously, it is caused by the unique distribution of energy dissipation rate. In the case of static mixers, where the profile is homogeneous, the combination of low *P_US_*, with higher *f_US_* = 59 kHz, provides the best results (the product most closely resembles the monodispersed one). Lower values of *f_US_* (here 40 kHz), at which bigger cavitation voids are formed, are responsible for a slight particles’ fragmentation, shown as a tail of the curve. In STR, the used *P_US_* was too low, so even the decrease of US frequency, up to the value of 40 kHz, would not give the desired result of agglomeration elimination. This confirms that the proper selection of ultrasonic power is substantial.

Taking into account the residence time (as well as sonication time—Figure 6, in the case of fast ionic reactions, such as CaF_2_ precipitation, one should avoid its extension and carry out the process in the shortest possible (from a practical application point of view) period of time. As shown, the extension of the residence time causes an increased number of crystal–crystal, as well as crystal–mixing equipment collisions, and leads to the increased destruction of solid grains. Such a conclusion is true for both examined reactor types; however, in the STR, the impact of the residence time seems to have a greater meaning (in comparison to Koflo STM), probably due to the much higher values of *τ*, compared to *t_m_*. Due to the same reason, in STR, one may obtain particles of a smaller dominant size. On the other hand, static mixers allow us to produce crystals with a smaller spread. It means that the number of particles whose size differs from the dominant value is less, in comparison to STR.

### 4.4. Dedicated Shape of Solid Particles (Cubes or Spheres)

The post-processing stages, carried out on the crystals obtained during crystallization/precipitation processes, often require a specific shape of crystals. Thin, rod-like, or needle-shape grains, elongated in one direction, are difficult to process, have rather poor flowability, and, in their original state, are inadequate for direct compression (i.e., during the production of tablets) [17]. Cubic crystals may easily be stacked to fill up all the empty space, and their compression results in the formation of the closest packed structures [18]. In turn, spherical crystals, after packing, always form structures with unfilled space (the percent of free space varies between 54 and 74% and depends on the arrangement of spheres) [18]. Such a space may be practically used, i.e., in the pharmaceutical industry, when, besides the active pharmaceutical ingredient, there should also be a place for auxiliary substances and fillers [17,19]. What is more, the particle size analyses, by the use of laser methods, are direct and easy, as no additional correction factors, taking into account the deviations of grains’ shape from spheres (so-called shape factors), have to be introduced to obtain reliable research results.

**Figure 8:**
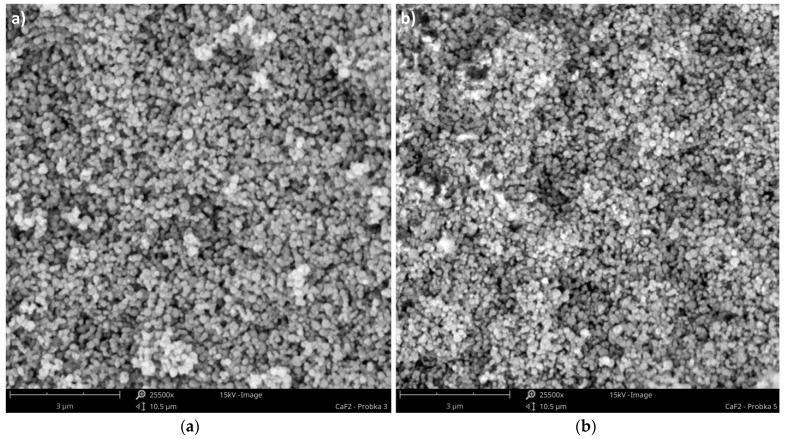
The shape of CaF_2_ particles precipitated in the Koflo STM—SEM images. Magnification 20,000×, size bare: 3 μm; (**a**) silent conditions *Re_eqv_* = 1091; (**b**) US-assisted system *Re_eqv_* = 6623.

During the research, both crystal shapes, namely cubic (i.e., natural form) and spherical, were precipitated. When static mixers were used as a crystallizer, particles of a final spherical shape were obtained, in both US-assisted and silent conditions, regardless of the turbulence intensity represented by *Re_eqv_*—Figure 8. As a result of the chemical reaction, cubic-like crystals were formed, but due to the combination of high turbulence intensity and high destruction forces (mainly crystal–crystal and crystal–static insert mechanical collisions, as well as shear stresses), their corners were damaged by the attrition (mechanical abrasion). Such a supposition is confirmed by the presented SEM photos (Figure 8), as some natural cubic crystals can be also found.

**Figure 9:**
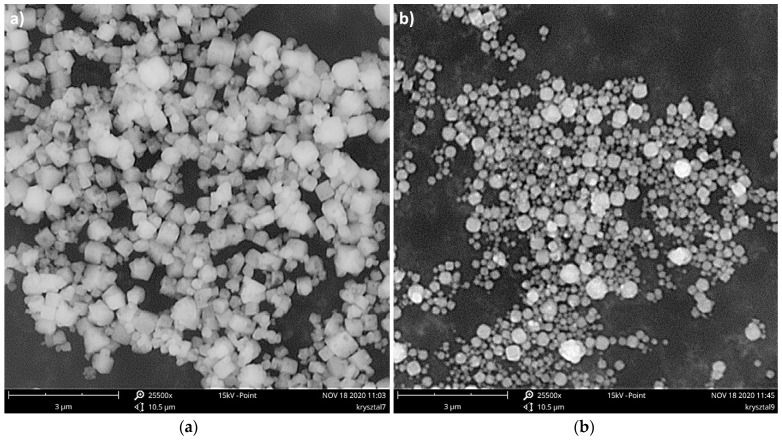
The shape of CaF_2_ particles precipitated in the US-assisted STR—SEM images (*ε_US_* = 14.9 W/kg). Magnification 25,500×, size bare: 3 μm; (**a**) *ε_mix_* = 1 W/kg; (**b**) *ε_mix_* = 3 W/kg.

If the precipitation carried out in the STR would be taken into account, one may observe that crystals of both shapes could be obtained—Figure 9. Cubic particles are common for a system working in silent conditions, unless the unit mixing power, *ε_mix_*, exceeds the limit at which the mechanical strength of single crystals would be decreased to such a point that particles’ attrition appears. Such a limit may, however, be impossible to reach in practice, as it would require a very high stirrer speed, especially for crystals with great toughness and hardness. During the research in silent conditions, for the stirrer’s unit power input in the range of *ε_mix_* = 0.4 ÷ 3 W/kg (i.e., 250 ÷ 500 rpm), the limit value has not been reached (the used *ε_mix_* values were insufficient even for agglomerates breaking), and the product had a cubic shape. In the US-assisted STR, the situation was similar. The combination of US assistance (*ε_US_* = 14.9 W/kg) and mechanical mixing gave the product a significantly reduced agglomerates number and a shape depending on the value of *ε_mix_*. For *ε_mix_* equal to 0.4 and 1 W/kg, cubic crystals were precipitated—Figure 9a. In turn, when *ε_mix_* was increased (up to 3 W/kg), spherical grains were formed as the destruction forces exceeded the strength of single particles, and the attrition began to be noticeable—Figure 9b.

### 4.5. Prediction of Crystal Size

**Figure 10:**
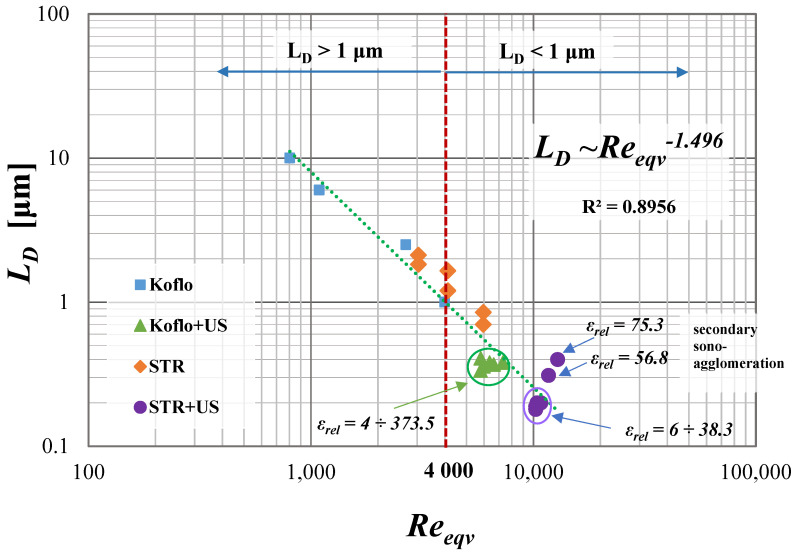
The dominant particle size *L_D_* as the function of equivalent Reynolds number *Re_eqv_* for all of the tested reaction systems.

One of the aims of the research carried out was to find a simple method that would enable the prediction of particle size. In previous authors’ papers [1,2], such attempts have been made before; however, the latest research allowed for its improvement. It was possible, due to the re-analysis of crystals obtained in STMs in silent conditions (as it was revealed, the collected plate-shaped structures, were, in fact, compressed agglomerated clusters made of small submicron particles), as well as the re-definition of STR equivalent diameter (as the primary one described the turbulence intensity as the same in the whole volume of the STR, which was not accurate, as the highest mixing intensity is present only in the nearest area of the agitator). After the corrections, a revised graphical method, Figure 10, was presented. By the use of such a method, one may see what can be expected if as a reaction system the STR or an in-line reactor with static inserts is used. One may also observe what would be the results of the additional support of ultrasounds. The method is based on the calculations of *Re_eqv_*, which are very simple and make the method available to everyone. The presented graphical representation also facilitates the planning stage of the experiment. If particles of a size greater than 1 μm are to be obtained, one should use the STM or the STR, both in silent conditions, and the equivalent Reynolds number should not exceed the limit value, which is 4000. In turn, if submicron particles are in demand, one may think of using the STR; however, the unit power input should be increased to the level in which *Re_eqv_* exceeds 4000. One may also consider the use of ultrasounds, which are responsible not only for the increase of turbulence intensity, but also in the case that STR could contribute to the alignment of the unit power distribution. However, one should bear in mind that the proper selection of US parameters is required, particularly when STR is used. Otherwise, especially when the ultrasonic power is selected incorrectly, one may expect that the agglomeration phenomenon will not be completely eliminated (if *P_US_* is too low) or that the particles’ fragmentation, hence secondary sono-agglomeration will occur (in the case of STR, for *ε_rel_* > 40).

It should be clearly indicated that the presented newly redeveloped method has been verified only for the precipitation of calcium fluoride CaF_2_ (*σ* = 157); therefore, the absolute values of the particles’ size may vary, depending on the crystals’ composition and mechanical strength. Despite the fact that the presented model is simplified, it provides a general view of the described research problem, presenting tips saying how one may change the particles’ characteristics, not only by the increase of turbulence in the system, but also by the selection of a reactor type, as well as the use of US-assistance. 

What could be also observed from the Figure 10 is that the sizes of the primary particles obtained by the use of STR are smaller than the one obtained in STM. The explanation lies in the turbulence intensity generated in the reactor. As presented, the fluid dynamic conditions in the STR are less favorable than the ones in STM in both cases, namely US-assisted and silent systems. In the STR, due to the use of a mechanical turbine stirrer or a combination of a mechanical stirrer with US, the turbulence intensity is greater (greater value of the equivalent Reynolds number); hence, the total energy destruction (described in detail in [4]) is higher. Due to that, the destruction is more harmful, and that leads to smaller particle sizes.

## 5. Conclusions

The presented paper, connected with CaF_2_ precipitation, shows comprehensive information on how to obtain a solid product with pre-determined characteristics.

The experimental studies took two types of reactors used into account: a Koflo static mixer (STM) and a stirred tank reactor (STR), both in silent and US-assisted systems.

The presented analysis of the research sheds new light on US-supported processes, as it revealed that the introduction of ultrasounds to the reaction system with a non-homogeneous *ε* distribution (like STR) may lead to the profile alignment and the change of mixing structure, depending on the relation between the unit power input of US and the unit power input of mechanical stirring, described by the authors as the relative input power value *ε_rel_*.

A significant and direct reduction of agglomerated structures during the precipitation process is possible when STR is used. However, it requires a high unit power of mechanical mixing *ε_mix_* if the system works under silent conditions (selected consciously and not to cause particles fragmentation) or with the use of US-assisted methods, with the proper selection of US generators localization and their parameters (bearing in mind the influence of cavitation voids on the generated crystals) is recommended. In turn, such an effect cannot be obtained when STMs are in use, as high compressive forces bind the particles (coated with a thin layer of a mother liquor) together.

To facilitate the post-processing of crystals, some might be interested in a monodispersed product. As shown, it is possible to obtain the population of crystals with a narrow CSD. The best results are observed when static mixers with the additional support of US are used as crystallizers; however, the use of US in the STR also leads to satisfactory results.

Taking into account the shape of the precipitated crystals, it is possible to obtain both spherical and cubic particles. Spherical ones are the result of high destruction forces, causing the attrition of cubes’ corners, and might be obtained by the use of STMs (in silent and US-assisted conditions, practically regardless of the turbulence intensity), and in STR with US, however, when *ε_mix_* ≥ 3 W/kg. In turn, cubic grains could be obtained in a silent STR or US-assisted STR, in which *ε_mix_* << 3 W/kg.

The comparison between the values of the nucleation intensity obtained in the STM and STR reactors shows that *B*_0_ was higher when the reactor with uniform *ε* distribution, namely the Koflo STM, was used. It is a reasonable observation, as the lack of weak mixing areas or dead zones caused the reaction between substrates taking place in the whole reactor volume. That, in turn, translates into high reaction efficiency and results in a high nucleation rate (in the analyzed range of *Re_eqv_*_,_ from 9·10^18^ to 7.9·10^19^). As the introduction of US (in the case of STMs) does not impact the *ε* distribution (it causes only the increase of turbulence), no significant changes in *B*_0*_rel_*_ were observed. The situation is completely different when STRs are taken into account. As silent STR is characterized by a very uneven distribution of unit power inputs, the nucleation rates could be improved by the intensification of turbulence (in the considered range of *Re_eqv_*_,_ from 2 × 10^17^ to 5.5 × 10^17^). However, if there are no contraindications, one can use US-assisted STR, in which the effects of ultrasonic waves would depend on the used ultrasonic frequency (low ultrasonic frequency, 20–100 kHz, is suggested for nucleation improvement) and the relative input power value *ε_rel_*. For *ε_rel_* lower than the limit (*ε_rel_* < 5), no significant effects of US were observed. For higher *ε_rel_* values, both the intensification of turbulence and the alignment of the *ε* profile by the introduction of US could be observed. Namely, if *ε_rel_* stays within the range of 5 to 35, then a moderate influence of US on *B*_0*_rel_*_ could be seen (50–60 times increase in *B*_0*_rel_*_). For *ε_rel >_* 35, the US impact will be dominant over the mixing, and the *ε* profile will tend to be homogeneous. That results in a substantial increase in ($B_{0_{rel}}\in$ 100 ÷ 10,000).

## 6. Patents

The patents describing the methods allowing us to obtain the micro-sized crystals of CaF_2_ with a narrow CSD and dedicated spherical shape are pending (patent applications P. 439332, P. 440602).

## Data Availability

The data used in the manuscript may partially be found in the previous authors’ papers, defined in the references section as [1,2,3,4]. New data are available from the corresponding authors upon reasonable request.

## Supplementary Materials

The following supporting information can be downloaded at: https://www.mdpi.com/article/10.3390/ma15217419/s1, Figure S1: The experimental setup; Figure S2: The influence of US power on particle density distribution *q*_3_ for both examined reactor types; Table S1: Properties of the reactants; Table S2: Dimensions of the used reactors; Table S3: Operating conditions of the precipitation process carried out in the Koflo STM for the constant value of *ε_mix_* = 0.1 W/kg; Table S4: Operating conditions of the precipitation process carried out in the Koflo STM for the constant value of *ε_US_* = 14.9 W/kg; Table S5: Operating conditions of the precipitation process carried out in the STR for the constant value of *ε_mix_* = 0.4 W/kg; Table S6: Operating conditions of the precipitation process carried out in the STR for the constant value of *ε_US_* = 14.9 W/kg.

## Nomenclature

$Re_{eqv}=\varepsilon^{\frac{1}{3}}\cdot d_{eqv}{}^{\frac{4}{3}}\cdot \frac{\rho }{\eta^{\prime }}$	equivalent Reynolds number
$d_{eqv_{STM}}=\sqrt{\frac{4\cdot V_{fs}}{\pi \cdot L_{m}}}$	equivalent diameter of a static mixer (m)
$d_{eqv_{STR}}=\sqrt[{3}]{V_{act}}$	equivalent diameter of a tank reactor (m)
$\varepsilon =\varepsilon_{mix}+\varepsilon_{US}$	total unit power input (W/kg)
$\varepsilon_{US}=\frac{P_{US}}{V_{UB}\cdot \rho }$	unit power input resulting from the ultrasounds’ use (W/kg)
$\varepsilon_{STM\;mix}=\frac{\dot{V}\cdot \Delta p}{V_{STM}\cdot \rho }$	unit power input resulting from mixing in the static mixer (W/kg)
$\varepsilon_{STR_{mix}}=X\cdot \frac{Ne\cdot d_{m}^{5}\cdot n^{3}}{V_{STR}}$	active unit power input resulting from mixing in STR (W/kg)
*B*_0_	nucleation rate (m^−3^s^−1^)
*B*_0*_rel_*_	relative nucleation rate (-)
*B*_0*_mix_*_	nucleation rate during experiments in silent conditions (m^−3^s^−1^)
*B*_0*_US_*_	nucleation rate during experiments with US-assistance (m^−3^s^−1^)
*CV*	coefficient of variation (%)
*D*	diameter of a tank crystallizer (m)
*d_act_*	active diameter of a device (m)
*d_eqv_*	equivalent diameter of a device (m)
*d_m_*	diameter of a mechanical stirrer (m)
*E*(*t*)	residence time distribution function (1/s)
*f_US_*	ultrasounds frequency (kHz)
*h_m_*	height of a mechanical mixer (m)
*k_v_*	crystals’ volume shape factor (-)
*L_D_*	dominant particle size (m)
*L_i_*	size of i^th^ crystal fraction (m)
*L_m_*	device length (m)
*L_mean_*	mean particle size (m)
*L*_16_	size of a crystal, for which Q_3_ equals 16% (m)
*L*_50_	size of a crystal, for which Q_3_ equals 50% (m)
*L*_84_	size of a crystal, for which Q_3_ equals 84% (m)
*ΔL_i_*	width of the i^th^ grain class (m)
*Ne*	power number (-)
*n*	number of stirrer revolutions (1/s)
*n*_0_	nuclei number density per unit volume (1/m^4^)
*n(L)*	population density (1/m^4^)
*P_nom_*	nominal power of US probe in laser particle analyzer (W)
*P_US_*	power of ultrasounds (W)
Δ*p*	pressure drop (Pa)
*Q*_3_	particle undersize distribution (%)
*q*_3_	particle density distribution (1/m)
*t*	time (s)
*t_m_*	residence time in STM (s)
$\dot{V}$	volumetric flow rate (m^3^/s)
*V_act_*	active volume of STR (m^3^)
*V_STM_*	volume of STM (excluding the static inserts) (m^3^)
*V_STR_*	volume of STR (m^3^)
*V_UB_*	volume of an ultrasonic bath (m^3^)
*w_i_*	mass fraction of crystals (kg/kg)
*X*	conversion factor, enabling the estimation of the ε_loc_ in the STR (Equation) (-)
*Y*	coefficient used for the evaluation of the active diameter in the STR (Equation), (-)
*Greek symbols*	
*ε*	total unit power input (W/kg)
*ε_rel_*	relative unit power input (W/kg)
*ε_loc_*	local unit power input (W/kg)
*ε_mix_*	unit power input resulting from mixing (W/kg)
*ε_US_*	unit power input resulting from the ultrasounds’ use (W/kg)
*η’*	fluid dynamic viscosity coefficient (Pa·s)
*ρ*	fluid density (kg/m^3^)
*σ*	relative supersaturation (-)
*τ*	residence time (s)
*τ_ind_*	induction time (s)
*ϕ*	volume fraction of crystals (m^3^/m^3^)
*ω*	number of stirrer revolutions (rpm)
*Subscripts*	
*act*	active
*av*	average
*eqv*	equivalent
*loc*	local
*STM*	static mixer
*STR*	stirred tank reactor
*Acronyms*	
*CFD*	computational fluid dynamics
*CSD*	crystal size distribution
*MSMPR*	mixed suspension mixed product removal
*RTD*	residence time distribution
*SEM*	scanning electron microscopy
*STM*	static mixer
*STR*	stirred tank reactor
*UB*	ultrasonic bath
*US*	ultrasounds
