# Supplementary material for: Solid Features Modification by the Reactor Selection and US Support during Reactive Crystallization
Source: Materials (Basel). 2022 Oct 22;15(21):7419. doi: 10.3390/ma15217419 (PMC9654633; doi:10.3390/ma15217419)
Supplement: Supplementary file 1 [file materials-15-07419-s001.zip › materials-1905968-supplementary.pdf]

SUPPLEMENTARY FILES

Table S1. Properties of the reactants

Property	Symbol with unit	NH_4F	$Ca(NO_3)_2$	Liquid-solid suspension	Mother liquor
Density	ρ [kg/m^3]	1003.14	1013.37	1009.35	1002.84
Viscosity	η [Pa·s]	$1.01 \cdot 10^{-3}$	$0.93 \cdot 10^{-3}$	nd[1]	$0.90 \cdot 10^{-3}$

Table S2. Dimensions of the used reactors

	Symbol with unit	Tank reactor	Koflo static mixer
Type of mixing operation	-	*mechanical*	*static*
Shape of mixing elements/mixer type	-	*turbine agitator*	*semi-circular inserts*
Number of mixing elements	*N*	-	*6*
Inner diameter of a reactor	*D [m]*	*0.07*	*0.015*
Diameter of mixing element	d_m *[m]*	*0.04*	*0.015*
Height of mixing element	h_m *[m]*	*0.012*	-
Length	*L [m]*	-	*0.186*
Height	*H [m]*	*0.07*	-
Total volume*	*V [mL]* ***[ml = 10^{-6} m3]***	*250*	*16.4* **excluding mixing inserts*
Additional equipment	-	*4 baffles height: 0.05 m width: 0.07 m*	-

[1] nd – not determined

Table S3. Operating conditions of the precipitation process carried out in the Koflo STM for the constant value of ε_{mix} = 0.1 W/kg

V [l/h]	t_m [s]	f [kHz]	ε_{mix} [W/kg]	Re_{eqv} (silent conditions)	P_{US} [W]	ε_{US} [W/kg]	$\varepsilon = \varepsilon_{mix} + \varepsilon_{US}$ [W/kg]	Re_{eqv}
40	1.61	40 i 59	0.1	1 091	150	14.9	15.0	5 790
					225	22.3	22.4	6 623
					300	29.7	29.8	7 287

Table S4. Operating conditions of the precipitation process carried out in the Koflo STM for the constant value of ε_{US} = 14.9 W/kg

V [l/h]	t_m [s]	f [kHz]	ε_{mix} [W/kg]	Re_{eqv} (silent conditions)	P_{US} [W]	ε_{US} [W/kg]	$\varepsilon = \varepsilon_{mix} + \varepsilon_{US}$ [W/kg]	Re_{eqv}
30	2.16	40 and 59	0.04	804	150	14.9	14.94	5 782
40	1.61		0.10	1 091			15.0	5 790
100	0.65		1.5	2 672			16.4	5 961
150	0.43		4.9	3 994			19.8	6 354

Table S5. Operating conditions of the precipitation process carried out in the STR for the constant value of ε_{mix} = 0.4 W/kg

τ [s]	f [kHz]	ω [rpm]	ε_{mix} [W/kg]	Re_{eqv} (silent conditions)	P_{US} [W]	ε_{US} [W/kg]	$\varepsilon = \varepsilon_{mix} + \varepsilon_{US}$ [W/kg]	Re_{eqv} (US + mix)
30 and 9	40 and 59	250	0.4	3 022	150	14.9	15.3	10 175
					225	22.3	22.7	11 613
					300	29.7	30.1	12 763

Table S6. Operating conditions of the precipitation process carried out in the STR for the constant value of ε_{US} = 14.9 W/kg

τ [s]	f [kHz]	ω [rpm]	ε_{mix} [W/kg]	Re_{eqv} *(silent conditions)*	P_{US} [W]	ε_{US} [W/kg]	$\varepsilon = \varepsilon_{mix} + \varepsilon_{US}$ [W/kg]	Re_{eqv} *(US + mix)*
30 and 9	40 and 59	250	0.4	3 022	150	14.9	15.3	10 175
		350	1	4 102			15.9	10 306
		500	3	5 916			17.9	10 722

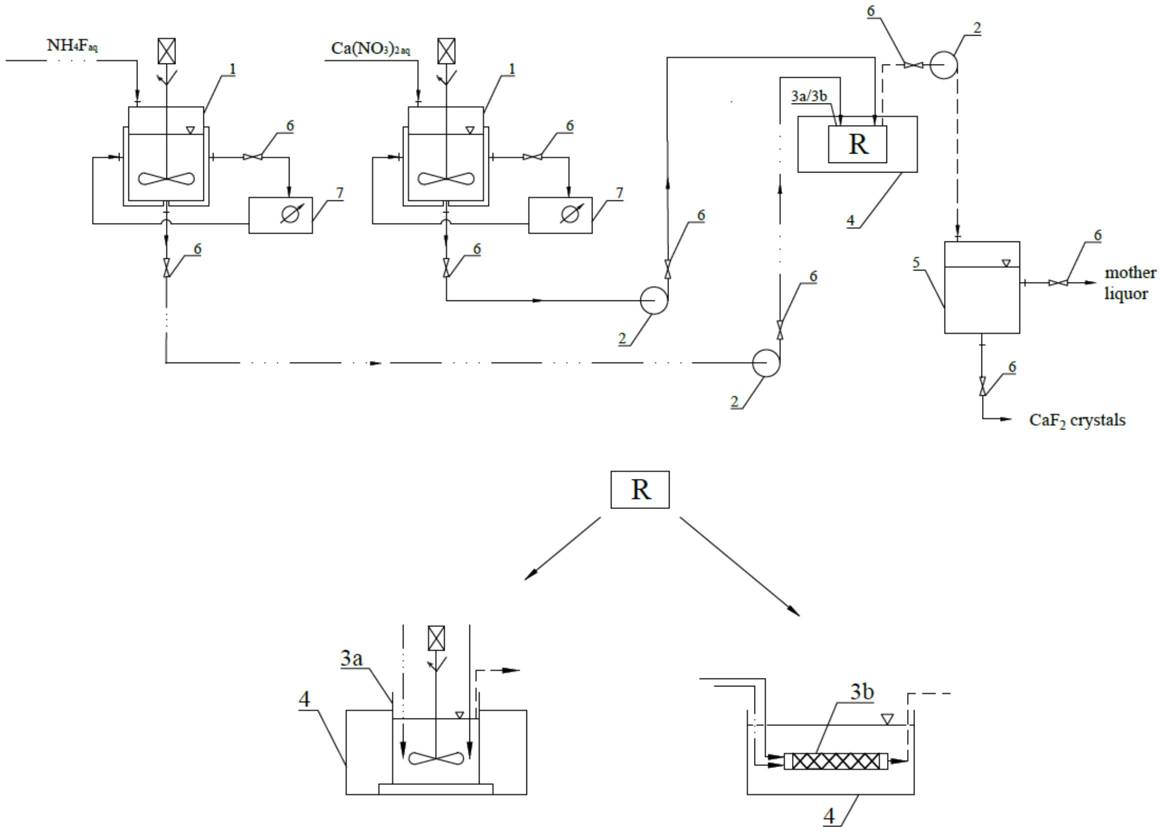

Figure S1. The experimental setup
1 – substrate tank with a mechanical stirrer, 2 – pump, 3a – STR with a turbine stirrer, 3b – static mixer, 4 – US bath, 5 – storage tank, 6 – valve, 7 – thermostat, R – reactor (3a, b)

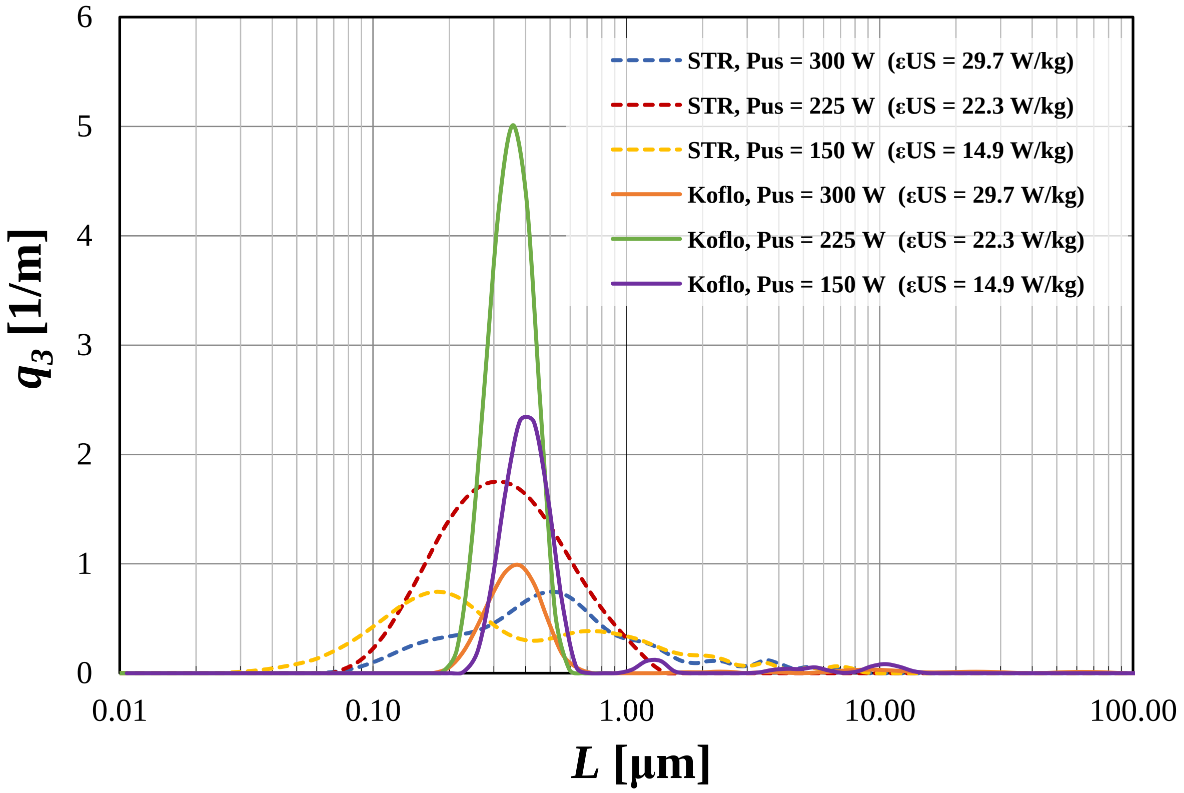

Figure S2. The influence of US power on particle density distribution q_3 for both examined reactor types
